# Sociodemographic Factors and Sex Education Sources: Potential Determinants of Adherence to Modern Myths About Sexual Aggression

**DOI:** 10.1192/j.eurpsy.2026.12110

**Published:** 2026-07-31

**Authors:** D. Triki, I. Baati, F. Guermazi, M. Abdelkafi, I. Feki, J. Masmoudi, R. Masmoudi

**Affiliations:** Psychiatry A, Hedi Chaker university hospital, Sfax, Tunisia

## Abstract

**Introduction:**

The perpetuation of modern myths about sexual aggression (AMMSA) is a barrier to gender equality and effective victim support. Young physicians, as future influencers of public health, are a critical population for studying the determinants of these beliefs.

**Objectives:**

To identify the sociodemographic factors and sources of information on sexuality associated with a higher adherence to (AMMSA) in a population of young physicians.

**Methods:**

We conducted a cross-sectional study among 153 young doctors in Sfax. Data were collected via an online questionnaire (sociodemographic, sexual knowledge sources, French version of the AMMSA scale). Univariate analysis (Student’s t-test, ANOVA and Spearman correlation) was used to compare AMMSA scores across different variables.

**Results:**

The mean global AMMSA score was 102.05 ± 24.67. Male participants had significantly higher scores (114.82 ± 28.13) than females (97.68 ± 21.84), with a p-value <10⁻³.

Participants from rural origin also showed greater adherence to myths (111.5 ± 9.11) compared to urban areas (101.52 ± 25.17, p=0.021). Furthermore, having children was associated with a higher AMMSA score (mean score=111.14 ± 16.78 vs 100.6 ± 25.45, p=0.019). A weak but significant positive correlation with age was also observed (rho=0.176, p=0.03).

Regarding information sources, participants who cited television as a source had significantly higher AMMSA scores (104.8 ± 25.61) than those who did not (95 ± 22.13, p=0.027). Sources such as parents, friends, and the internet showed no significant association.

**Conclusions:**

This study identifies male gender, rural origin, parenthood, and reliance on television as a source of sexual information as key factors associated with a stronger belief in modern myths about sexual aggression. Public health interventions aimed at deconstructing these myths among young professionals should be tailored to target these specific populations and actively counter the misleading narratives often perpetuated by mainstream media.

**Disclosure of Interest:**

None Declared

